# Brain-Derived Neurotrophic Factor in Brain Disorders: Focus on Neuroinflammation

**DOI:** 10.1007/s12035-018-1283-6

**Published:** 2018-08-17

**Authors:** Bruno Lima Giacobbo, Janine Doorduin, Hans C. Klein, Rudi A. J. O. Dierckx, Elke Bromberg, Erik F. J. de Vries

**Affiliations:** 1 Neurobiology and Developmental Biology Laboratory, Faculty of Biosciences, Pontifical Catholic University of Rio Grande do Sul, Ipiranga Av. 6681, Porto Alegre, 90619-900 Brazil; 2 Department of Nuclear Medicine and Molecular Imaging, University of Groningen, University Medical Center Groningen, Hanzeplein 1, P.O. Box 31.001, 9713 GZ Groningen, The Netherlands

**Keywords:** Brain-derived neurotrophic factor, Neuroinflammation, Neurological disorders, Neurotoxicity

## Abstract

Brain-derived neurotrophic factor (BDNF) is one of the most studied neurotrophins in the healthy and diseased brain. As a result, there is a large body of evidence that associates BDNF with neuronal maintenance, neuronal survival, plasticity, and neurotransmitter regulation. Patients with psychiatric and neurodegenerative disorders often have reduced BDNF concentrations in their blood and brain. A current hypothesis suggests that these abnormal BDNF levels might be due to the chronic inflammatory state of the brain in certain disorders, as neuroinflammation is known to affect several BDNF-related signaling pathways. Activation of glia cells can induce an increase in the levels of pro- and antiinflammatory cytokines and reactive oxygen species, which can lead to the modulation of neuronal function and neurotoxicity observed in several brain pathologies. Understanding how neuroinflammation is involved in disorders of the brain, especially in the disease onset and progression, can be crucial for the development of new strategies of treatment. Despite the increasing evidence for the involvement of BDNF and neuroinflammation in brain disorders, there is scarce evidence that addresses the interaction between the neurotrophin and neuroinflammation in psychiatric and neurodegenerative diseases. This review focuses on the effect of acute and chronic inflammation on BDNF levels in the most common psychiatric and neurodegenerative disorders and aims to shed some light on the possible biological mechanisms that may influence this effect. In addition, this review will address the effect of behavior and pharmacological interventions on BDNF levels in these disorders.

## Introduction

Brain disorders are among the major causes of disability and morbidity worldwide. According to recent projections, the incidence of such diseases will increase in the next decades [1]. The lack of adequate treatment turns these diseases into a significant problem worldwide, and the absence of effective treatment can partly be ascribed to our incomplete knowledge of the etiology of most brain disorders. Many mental diseases, however, are tightly associated with environmental stimuli, such as stress [2]. Challenging events can pose a significant burden on individuals, especially those more sensitive to its effects. Although acute stress can have benefits (e.g., enhanced attention, memory), it can also become life threatening when stressful events become a routine part of the life of individuals. A number of studies have already shown that stress is associated with metabolic changes, cardiovascular risk, endocrine abnormalities, mood changes, and impairment of cognitive functions (i.e., mild cognitive impairment), leading to an increased risk of developing psychiatric and neurologic disorders [2, 3]. Chronic stress can lead to the activation of pro-inflammatory microglia, releasing cytokines and pro-inflammatory substances, and recruitment of peripheral immune cells to the brain, thus creating the inflammatory environment that is characteristic for many brain pathologies.

In order to cope with stressful events, brain cells release several substances that can promote neuronal survival, such as antiinflammatory cytokines, growth factors and neurotrophic factors. One of the best-studied neurotrophins is the brain-derived neurotrophic factor (BDNF). Brain pathologies are usually associated with a downregulation of BDNF release, resulting in reduced BDNF levels in the brain and in blood. BDNF has been suggested as a candidate biomarker of pathological conditions, and therapy efficacy, as most of current treatments are accompanied by a significant change in blood BDNF levels. However, there is still a gap in our understanding of the physiological mechanisms that lead to changes in BDNF levels under pathological conditions.

This review will summarize our current knowledge of BDNF in the pathophysiology of the most common brain disorders. Since neuroinflammation has been considered an important mediator for the onset and progression of many brain pathologies, this review will also attempt to explore the interaction between neuroinflammation and BDNF expression in the brain.

## BDNF Expression and Function

**Fig. 1:**
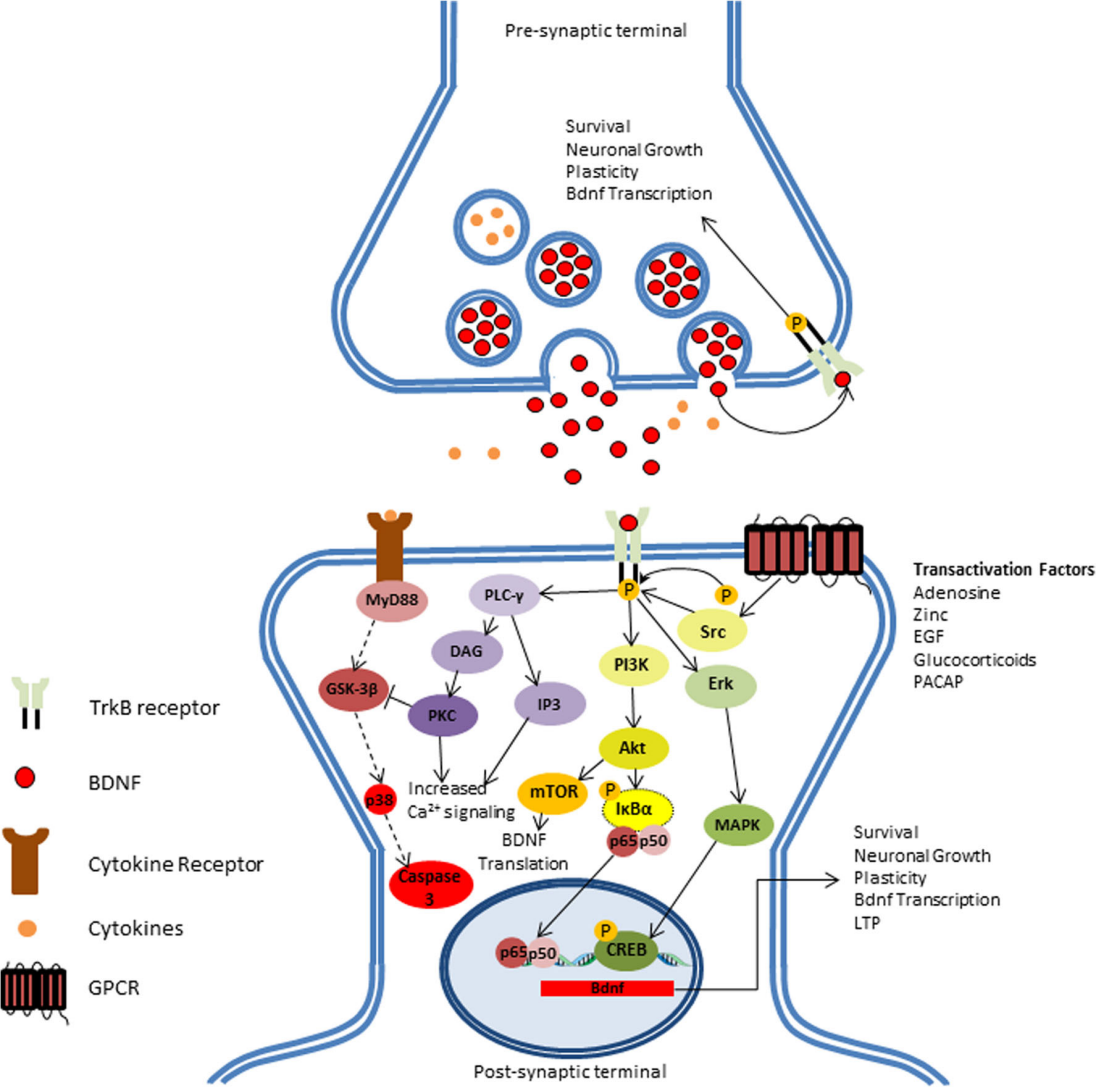
BDNF induces survival-related signaling mechanisms: BDNF induces survival-related signaling mechanisms: In physiological conditions, binding of BDNF to TrkB receptor in either paracrine or autocrine signaling elicits three distinct downstream pathways. BDNF-dependent phospholipase C-gamma (PLC-γ) can induce short-term signaling by increasing Ca2+ neuronal response and inhibit inflammatory-dependent apoptosis cascade (dashed lines) by inhibition of glycogen synthase kinase 3-beta (GSK-3β). Induction of phosphatidylinositol 3-Phosphate (PI3K) induces transcription of BDNF mRNA by activating mTOR-dependent translation of BDNF. Additionally, BDNF can modulate gene regulation by activating NF-κB and CREB transcription factors by inducing Akt and Erk downstream pathways, respectively. Gene modulation induces neuronal survival, growth, long-term potentiation (LTP), and de novo expression of BDNF. In addition, BDNF-independent transactivation of TrkB can also play an important role in the neurotrophic pathway regulation by factors, such as adenosine, zinc, epidermal growth factor (EGF), glucocorticoids, and pituitary adenylate-cyclase-activating polypeptide (PACAP), further enhancing TrkB signaling in the synapse

BDNF is a member of the neurotrophin family, which also includes neural growth factor (NGF) and neurotrophins 3 and 4. The Bdnf gene is comprised of a common 3′-exon that encodes the pro-BDNF region of the protein, and several species-dependent 5′-noncoding, promoter-regulated regions, terminating in a coding 5′-exon that encompasses the gene expression [4, 5]. Bdnf gene expression is strongly regulated by a wide array of endogenous and exogenous stimuli (e.g., stress, physical activity, brain injury, diet). BDNF is translated as a pro-neurotrophin (pro-BDNF) that can be cleaved into mature BDNF in the cytoplasm by endoproteases or in the extracellular matrix by plasmin or matrix metalloproteinases (MMP). Both mature BDNF and pro-BDNF can be secreted and bind to the low affinity p75 neurotrophin receptor (p75NTR), which causes activation of the apoptosis cascade [6, 7]. On the other hand, cleaved, mature BDNF binds to its high-affinity receptor tyrosine kinase B (TrkB), activating several signaling cascades, including the Ras-mitogen-activated protein kinase (MAPK), the phosphatidylinositol-3-kinase (PI3K), and the phospholipase Cγ (PLC-γ) pathway. These signaling cascades induce an increase in Ca^2+^ intake, phosphorylation of transcription factors, and de novo expression of the Bdnf gene (Fig. 1) [8]. Although pro-BDNF can act as a signaling factor for the apoptotic cascade, it is not yet clear if pro-BDNF is secreted by neurons under normal conditions as its concentration in presynaptic terminals is relatively low when compared to mature BDNF. Indeed, in animal models, the concentration of mature BDNF can be ten times higher than the concentration of pro-BDNF [9, 10], which poses a question regarding the efficacy of pro-BDNF as a proper signaling factor.

BDNF has a wide array of functions within the brain and is highly abundant in several brain structures. In the brain, BDNF is involved in plasticity, neuronal survival, formation of new synapses, dendritic branching, and modulation of excitatory and inhibitory neurotransmitter profiles [11, 12]. BDNF is active at all stages of development and aging [13]. Knockout mice lacking BDNF rarely reach adulthood and, when they do, there is a development of several sensory impairments [14, 15]. BDNF is also found in peripheral organs, such as the heart, gut, thymus, and spleen [16, 17]. Around 90% of the BDNF in blood is stored within platelets [18]. Many brain pathologies cause reduction of BDNF protein levels both in the brain and serum of patients [19–22]. Unfortunately, it is still unclear whether BDNF protein levels measured in serum samples reflect BDNF levels in the brain, as studies in animal models gave contradictory results so far [23–25].

### BDNF in Neuroinflammation

**Fig. 2:**
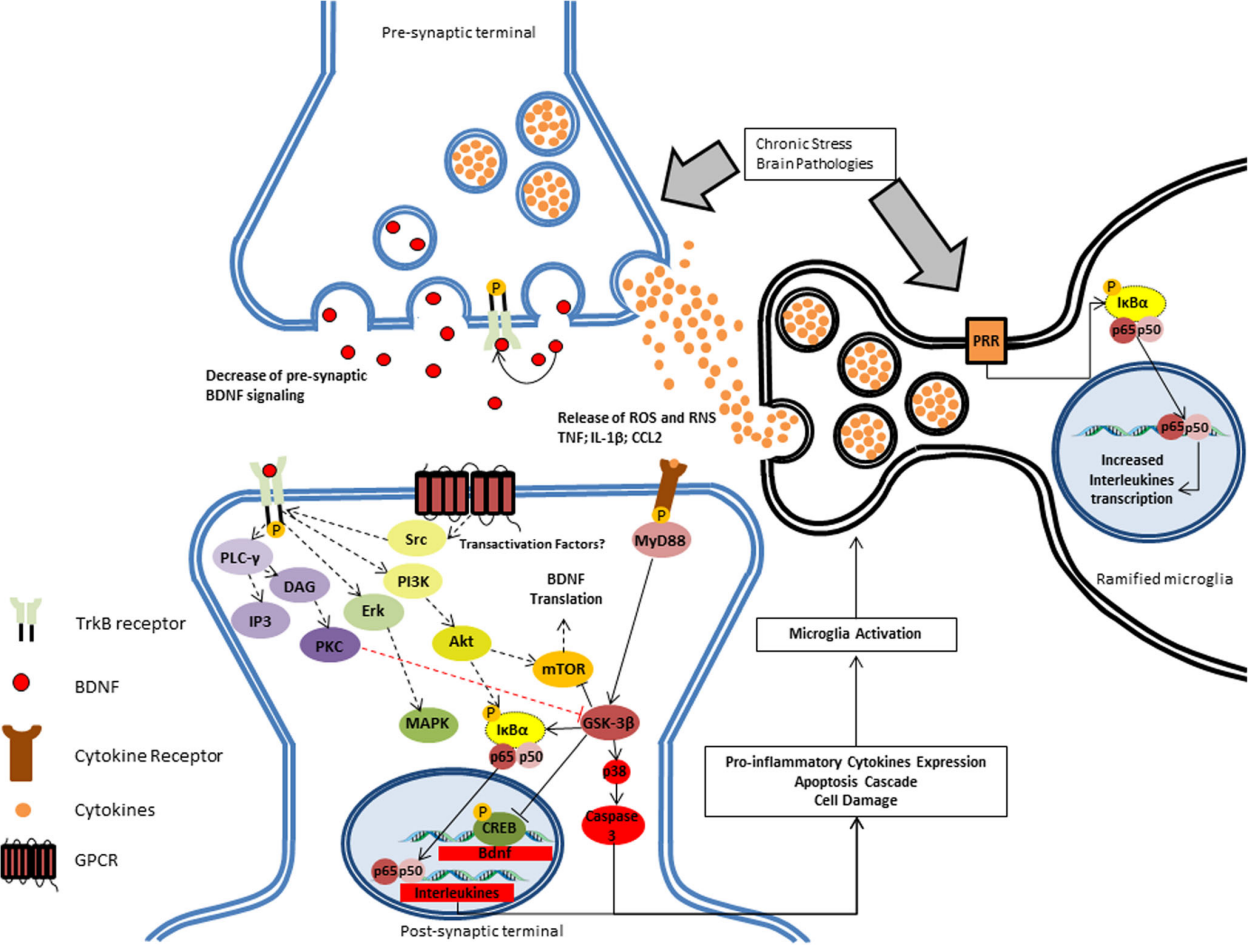
BDNF response after inflammatory brain pathogenicity. In chronically stressful situations, such as brain pathologies, there is an induction of NF-κB-dependent pro-inflammatory activation of microglia after induction of the pattern recognition receptor (PRR) by the challenge (e.g., stress or pathology). Pro-inflammatory cytokines, especially IL-1, can directly bind microglial cells, which result in induction of the expression and release of several mediators, most of which are neurotoxic, including reactive oxygen (ROS) and nitrogen species (RNS), pro-inflammatory cytokines (such as tumor necrosis factor), and chemokines (such as CC-chemokine ligand 2, CCL2; also known as MCP1). Additionally, there is a decrease of BDNF signaling in the synaptic cleft, further reducing BDNF-dependent survival-related signaling (black dashed lines) and inhibition of apoptotic pathways, such as glycogen synthase kinase 3-beta (GSK-3: red dashed line). Such factors will lead to an increase of NF-κB complex binding to genes that express pro-inflammatory cytokines (e.g., interleukin 1-β, IL-6, IL-8, TNF). The effect of transactivation factors on the BDNF-independent maintenance of TrkB is not clear yet

After inflammatory signaling (e.g., stress, pro-inflammatory signals), several signaling cascades are changed within the cell. This signaling generates a sequence of events that may eventually lead to neuronal malfunction and apoptosis. Microglia also participate actively in the development of pathological neuroinflammatory process by releasing pro-inflammatory cytokines, which contribute to the neurotoxicity. This cycle is repeated as long as the stressor is present, which can develop into serious consequences (e.g., cognitive impairment, behavioral dysfunction, neurological and psychiatric disorders). One of the main factors of inflammatory activation is the nuclear factor-kappa B (NF-κB), a transcription factor that induces the expression of several pro- and antiapoptotic genes, including Bdnf [26]. Interestingly, binding of BDNF to the TrkB receptor can also induce the expression of NF-κB, although the pathways for this modulation are yet unclear (Fig. 2). NF-κB is closely involved in the innate and adaptive immune response in several psychiatric and neurodegenerative diseases [27]. NF-κB is a regulator of, e.g., apoptosis, neuronal survival and proliferation, and migration and maturation of immune cells [28]. BDNF-induced NF-κB expression stimulates PLC-γ/PKC signaling through the activation of the kinases IKKα and IKKβ. These kinases phosphorylate the NF-κB inhibitory unit IκBα, resulting in the binding of ubiquitin and subsequently degradation of IκBα by proteasomes [29]. IκBα degradation induces the release of the NF-κB and formation of the p50/p65 dimer, which binds to the DNA and induces the expression of genes related to neuronal proliferation, survival, and inflammatory response [29, 30]. Furthermore, it is known that BDNF can also bind to the p75NTR. Even though the affinity of BDNF for the p75NTR receptor is several times lower than for TrkB [31], a p75NTR-mediated effect on NF-κB expression can be observed. Studies have shown that activation of p75NTR increases apoptotic and inflammatory signaling in neurons and glial cells by activation of c-Jun N-terminal kinases (JNK) and NF-κB expression, respectively [32, 33]. However, the effect the p75NTR has on neurotrophic signaling is still under debate, as there is no clear evidence on how large the role of the p75NTR is in mediating such processes.

Thus, the role of BDNF in neuroinflammation is strongly related to its ability to induce—and being induced by—NF-κB. However, the exact regulatory mechanisms are not yet clear.

## BDNF and Aging

The aging process can lead to the impairment of several brain functions. Studies have reported a decrease in whole brain volume in elderly when compared with young adults, especially in brain regions related to cognition [34–37]. Upon aging, microglia increasingly adopt a pro-inflammatory state due to a decrease in the resting signaling by neurons and astrocytes [38, 39]. As a result, external stimuli (e.g., stress, trauma, infection) can submit the aged brain more easily into a state of mild chronic neuroinflammation, making the brain more prone to apoptotic signaling [40]. This can lead to volume loss and the associated cognitive impairment [41]. Animal studies in stress models, such as chronic stress, maternal separation, and social defeat, have confirmed that stress is associated with glial activation and that aging decreases cognitive function [42, 43]. The loss of volume is indicative of a reduction in the global neuronal network, and consequently a diminished brain plasticity that could support the brain in such events, thus reducing the cognitive function [44, 45].

It is known that BDNF plays a role in maintaining brain function by inducing survival signaling and neuroplasticity. Although the effect is more visible in younger population, elderly subjects also benefit from it, especially those cognitively, physically, and socially active, reducing the risk of age-related comorbidities [46]. Studies in animal models report an increase in brain BDNF levels when aged animals are submitted to protocols such as long-term environmental enrichment [47–49] or physical activity [50, 51]. Studies on aged mice demonstrated that animals heterozygous for BDNF showed decreased fear extinction learning [52] and conditioned fear learning [53] when compared with young heterozygous mice, but the mechanism responsible for the behavioral effects is not completely clear yet. Better understanding of the processes that are modulated by BDNF may facilitate the development of novel therapeutic methods that could help to prevent or counteract the aging effects on the human brain.

## BDNF in Psychiatric Disorders

Whenever the brain is challenged by harmful events, its coping mechanisms are activated in order to revert the system to homeostasis. When these coping mechanisms fail, e.g., due to excessive damage, enhanced sensitivity, and chronic or recurrent exposure to stimuli, a disturbance of normal brain function may occur that can lead to the onset of neuropsychiatric disorders (Table 1). Although psychiatric diseases can display wide spectra of symptoms, common phenomena in these disorders are a disarray of excitatory/inhibitory neurotransmitter signaling and loss of neuronal function, leading to mood and behavioral disturbances, and cognitive impairment.

**Table 1:** Effects of BDNF on several neuropsychiatric and neurodegenerative conditions

Condition	Peripheral BDNF	Brain BDNF	References
Major depressive disorder (MDD)	Decreased serum and plasma levels of BDNF protein; some literature findings showing no change or increased levels in MDD patients; euthymic patients have normalized BDNF levels in serum or plasma; increased methylation of the Bdnf gene is associated with a decrease in mRNA expression; treatment-resistant patients show lower BDNF levels in serum compared to treatment-responsive patients	Decreased BDNF and TrkB mRNA expression in hippocampal slices of MDD patients; use of antidepressant medication was associated with increased Bdnf mRNA expression	Elfving et al. (2012), Karege et al. (2005), Matrisciano et al. (2009), Pandey et al. (2008), Thompson Ray et al. (2011), Hong et al. (2014)
Bipolar disorder (BD)	Decreased serum and plasma levels of BDNF in both manic and depressive stages of BD; euthymic patients show no difference from controls	Decreased BDNF mRNA expression in hippocampus of suicidal BD patients; no difference in Bdnf expression between different disease stages (euthymic, depressive or manic)	Banerjee et al. (2013), Knable et al. (2004), Monteleone et al. (2008), Sklar et al. (2002), Ray et al. (2014)
Schizophrenia (SCZ)	Decreased BDNF protein levels in serum of SCZ patients; no changes in BDNF levels after treatment	Decreased expression of Bdnf and Trkb genes in hippocampus and dorsolateral prefrontal cortex of SCZ patients; increased methylation of Bdnf gene in prefrontal cortex of SCZ patients	Dong et al. (2015), Pillai (2008), Rao et al. (2015), Reinhart et al. (2015), Rizos et al. (2008), Weickert et al. (2003), Weickert et al. (2005), Xiu et al. (2009), Zhang et al. (2012)
Alzheimer disease (AD)	Low serum BDNF levels correlate with development of dementia—especially AD; decreased levels of BDNF in serum of AD patients; BDNF levels are not related to severity of disease; successful treatment transiently increases BDNF in AD	BDNF genotype is related with reduced Hippocampal activity and cognitive function in subjects with high levels of A-β and AD patients; decreased BDNF mRNA levels in the hippocampus of AD patients; increase in methylation pattern of the Bdnf gene in the frontal cortex of AD patients	Buchman et al. (2016), Honea et al. (2013), Lee et al. (2005), Lim et al. (2014), Phillips et al. (1991), Rao et al. (2012), Weinstein et al. (2014)
Parkinson disease (PD)	Serum BDNF levels are directly correlated with degeneration of striatum in PD; low serum levels of BDNF is correlated with decreased cognitive function in early PD patients; BDNF decrease in serum is associated with the progression of motor symptoms	Low BDNF mRNA expression in the striatum of PD patients; association between BDNF polymorphism and disease progression	Cagni et al. (2016), Howells et al. (2000), Scalzo et al. (2010), Y. Wang et al. (2016), Ziebell et al. (2012)
Epilepsy	BDNF val66met single nucleotide polymorphism is associated with higher BDNF protein expression and an increased risk of developing epilepsy; increased serum BDNF protein levels after epileptic seizures are associated with increased glutamate signaling	Increased mRNA expression of Bdnf exons in the hippocampus and cortex of temporal lobe epilepsy patients; increased BDNF protein expression in the hippocampus of temporal lobe epilepsy patients	Brooks-Kayal et al. (2009), Kandratavicius et al. (2013), Martínez-Levy et al. (2016), Martínez-Levy et al. (2017), N. et al. (2016), Warburton et al. (2016)

In humans, BDNF is known to be a useful biomarker for several psychiatric disorders [54]. Most chronic psychiatric diseases are accompanied by changes in BDNF levels, but it is still unclear if changes in BDNF levels are the cause or the result of the disturbances of normal brain function. Therefore, the effects of BDNF levels have been investigated in animal models. Heterozygous BDNF mice show increased weight gain, aggressiveness, anxiety, and contextual memory impairment and therefore have been suggested as an animal model for mood disorders [55]. These results suggest that BDNF could be a key player in the development of several symptoms associated with psychiatric disorders. This hypothesis is supported by the fact that effective treatment of these symptoms resulted in normalization of BDNF levels [56]. In this section, we will further discuss the role of BDNF in the main psychiatric disorders: major depressive disorder, bipolar disorder, and schizophrenia.

### Major Depressive Disorder

Major depressive disorder (MDD) is a common psychiatric disease characterized by abnormal behavior, anhedonia, sleep and dietary problems, cognitive impairment, and, in more severe cases, suicidal tendencies. Biologically, depression is related to a decrease of neurotransmitter signaling in the brain, dysfunction of hypothalamus pituitary adrenal axis (HPA-axis), increase in inflammatory signaling, and reduction in hippocampal volume.

Both MDD patients [57–59] and animal models of depression [60, 61] show a remarkable reduction in serum BDNF levels. Karege and colleagues have shown that this decrease is not related to platelet-associated BDNF release in the bloodstream [62], suggesting that reduced BDNF levels in the brain rather than a reduction in the peripheral release of BDNF by platelets are the cause of altered protein levels in blood. The magnitude of the decrease in plasma BDNF levels is associated with disease duration [63], but it is not clear yet whether the severity of symptoms is related to BDNF levels. BDNF single nucleotide polymorphism (val66met), however, is associated with the severity of depression in patients [64]. Successful antidepressant treatment is usually associated with an increase in BDNF levels in serum and plasma [65, 66], whereas treatment failure is associated with a lack of response of plasma BDNF levels. Thus, BDNF seems an important player in the pathophysiology and might be a biomarker for monitoring treatment response in depression [65, 67]. Epidemiological studies show that a third of all MDD patients experience no changes in the symptoms when treated with the most commonly used antidepressants. Postmortem studies revealed that these treatment-resistant patients have significantly lower BDNF levels, especially in BDNF-rich brain structures, such as the hippocampus [68–70]. Treatment with the rapidly acting antidepressant ketamine was able to increase plasma BDNF levels to the level of healthy controls [71, 72]. Ketamine is an inhibitor of NMDA receptors, inducing rapid, glutamate-dependent Ca^2+^ signaling and activation of cAMP response element-binding protein (CREB). Clearly, there is a need to increase our knowledge on the role of BDNF in MDD.

Induction of a pro-inflammatory response by systemic application of lipopolysaccharide (LPS) causes depressive-like symptoms (i.e., sickness behavior) in rodents [73, 74], which may also affect BDNF levels. Increased pro-inflammatory signaling leads to a reduction in the mRNA expression of Bdnf and other neurotrophins in plasticity-related brain structures, especially cortical regions [75]. The effects of reduced BDNF expression levels in mice, treated with a systemic LPS injection, can be counteracted by induction of TrkB-mediated signaling with the agonist 7,8-dihydroxyflavone, which leads to a reduction of depressive-like behavior [76]. Gibney and colleagues have found increased expression of interleukins IL-1β, IL-6, and tumor necrosis factor-α (TNF-α) and reduced expression of Bdnf genes in depressive-like rats 6 h after an inflammatory challenge. In this study, expression of cytokines returned to baseline levels after 48 h, but Bdnf mRNA remained low in frontal cortex and hippocampus [77]. In humans, chronic stress is one of the main precursors of depressive symptoms [78, 79]. In laboratory stress-conditioning, depressed patients show a higher inflammatory response, characterized by increased IL-6 release and NF-κB DNA binding, than healthy controls [80]. Depressive symptoms can also be caused by treatment that stimulate the immune system, such as interferon-α (IFN-α). Patients treated with IFN-α were shown to have decreased serum BDNF levels in combination with increased protein levels of the cytokines IL-1 and IL-2 [81, 82]. Interestingly, individuals that had higher BDNF levels at baseline showed better resilience to IFN-α-induced MDD.

These preclinical and clinical findings show that long-term exposure to stress or inflammation leads to a decrease in BDNF levels, reducing the capacity of the neurons to cope with further challenges (i.e., neuronal plasticity), and ultimately leading to a decreased function and neuronal death. Interestingly, treatment with antidepressants can result in an antiinflammatory response throughout the brain, mitigating the inflammatory unbalance to homeostatic levels and normalizing BDNF concentrations [83, 84]. However, further research is needed to understand the mechanisms involved in the regulation of BDNF by neuroinflammation.

### Bipolar Disorder

Bipolar disorder (BD) is characterized by fluctuations of mood throughout lifetime, oscillating between depressive, euthymic, and manic episodes. BD is associated with cognitive impairment and other comorbidities that affect the quality of life of the individual [85–87]. BD is characterized by alterations in dopaminergic and glutamatergic neurotransmitter systems, mitochondrial dysfunction, and increased oxidative stress, which in turn are related to neuroinflammation, neurotoxicity and eventually neuronal death [87]. Two recent meta-analyses have shown that serum and plasma levels of BDNF in BD patients during depressive and manic episodes are decreased, but no difference in BDNF levels between BD patients in an euthymic episode and healthy controls was found [88, 89]. It is known that treatment with mood stabilizers increases BDNF levels in prefrontal cortex and hippocampus of animals by inducing promoter IV-driven expression [90, 91]. Also in humans, treatment for the manic or depressive phases of BD is associated with an increase in serum BDNF levels [92, 93].

Recent studies have shown an association between in manic and depressive stages of BD and a pro-inflammatory profile of immune cells [94, 95]. Steiner and colleagues have found that suicidal mood disorder patients had a significant increase of microglial cell density in the dorsolateral prefrontal cortex, anterior cingulate gyrus, and mediodorsal thalamus compared to healthy controls and non-suicidal, mood disorder patients [96]. The presence of immune cells clusters in these brain regions suggests a strong inflammatory response, which could trigger the suicidal predisposition of these patients [96]. Although plenty of literature is available on BDNF or neuroinflammation in BD, there is a severe lack of studies regarding the association between both mechanisms on BD. Only two studies have analyzed both BDNF and cytokine levels in BD patients, with somewhat different conclusions. Patas and colleagues have shown an association between both serum BDNF and plasma IL-6 levels with a depressive episode associated with melancholic trait [97], while Wang and colleagues have found an association for serum BDNF levels, but not for IL-1β or IL-6 [98]. Clearly, more studies are needed to elucidate the interaction between neuroinflammation, BDNF, and disease symptoms in BD.

Interestingly, the most commonly used therapeutic drugs—lithium and valproate—were able to reverse the inflammatory state in mood disorders [99–101]. The most common assumption is that lithium and valproate can inhibit glycogen synthase kinase 3 (GSK-3) and sodium channel function, respectively [102]. Inhibition of GSK-3 activity by lithium increases cellular levels of BDNF. GSK-3 can inhibit mammalian target-of-rapamycin (mTOR)—an important modulator of BDNF-dependent neuronal plasticity and survival—and thus affect proper BDNF signaling and impair optimal cellular function. In the manic phase of BD, there is a remarkable increase in PKC-mediated signaling, which is associated with BDNF-dependent Ca^2+^ induction. PKC isozymes are involved in the pro-inflammatory response mediated by macrophages [103] and, more recently, microglia [104] through activation of the NF-κB inflammation pathway. Treatment of BD patients with lithium or valproate inhibits PKC upregulation, normalizing its level to that of euthymic subjects, and increases BDNF levels [105]. PKC inhibitor tamoxifen enhances the capacity of lithium to reduce symptoms of mania in BD [105–107]. PKC inhibition probably suppresses the expression of NF-κB and consequently resolves the NF-κB-mediated inhibition of Bdnf expression, resulting in an increase in peripheral BDNF levels in BD. However, the mechanisms underlying the increase in BDNF levels in response to treatment should still be further investigated. Yet, current evidence suggest that a decrease in BDNF levels can be considered as a biomarker for both depressive and manic stages of BD.

### Schizophrenia

Schizophrenia is a disease characterized by disturbances in the proper perception of a person’s surroundings. Schizophrenia is associated with a high suicide rate and accounts for a large number of hospitalizations, causing a significant burden to healthcare systems worldwide. The symptoms of schizophrenia comprise positive (e.g., hallucinations, delusions, confused thoughts, concentration impaired) negative (e.g., depression, anhedonia, self-neglect) and cognitive effects (e.g., memory, attention, reason impairments). Schizophrenic patients exhibit a decreased activation of γ-aminobutyric acid (GABA) signaling [108], inducing impaired neuronal activation, especially in dopaminergic neurons [109]. The etiology of schizophrenia is not fully understood yet and symptoms can vary between individual patients, making the diagnosis of schizophrenia challenging.

A recent meta-analysis revealed that serum BDNF levels in both drug-naïve and medicated schizophrenic patients are reduced. Serum BDNF levels in schizophrenic patients decrease with age but were independent of the dosage of medication [110]. However, it remains unclear if and how BDNF levels in the brain are altered in schizophrenic patients. Some studies report increased BDNF levels in frontal and temporal structures [111, 112], while others report decreased levels in the same brain structures [108, 113, 114]. Besides clinical observations, in vitro studies using the phencyclidine (PCP) psychosis model also give ambiguous results. Adachi and colleagues reported that exposure of cortical cultures to the non-competitive NMDA agonist PCP initially resulted in an increase in BDNF levels, whereas TrkB, ERK1/2, and Akt signaling were decreased [115]. In contrast, two other studies reported decreased BDNF mRNA expression in cortical slices after exposure to a low dose of PCP [116]. Taken together, in vitro, in vivo and clinical results seem to indicate that plasma BDNF levels are decreased in schizophrenic patients, but data on brain BDNF levels are contradictory.

Schizophrenia is a multifactorial disease, in which both genetic and environmental factors play a role. It is well established that physical and mental distresses can trigger psychotic behavior in (genetically) vulnerable patients [117]. These triggers can induce inflammatory changes that are associated with reduced neurotransmitter signaling, increased oxidative stress, and reduced synaptic branching [118]. Mondelli and colleagues have shown in leukocytes of first-episode schizophrenic patients that childhood trauma and the number recent stressful life events were negatively correlated with BDNF mRNA levels. BDNF levels were also negatively correlated with IL-6 expression, suggesting an inflammation mediated decrease in BDNF expression, or vice versa. Moreover, BDNF, IL-6, and cortisol levels correlated inversely with hippocampal volume [119]. A postmortem study demonstrated that schizophrenic patients have an increased inflammatory profile in dorsolateral prefrontal cortex. The group of patients with high levels of neuroinflammation had lower expression of BDNF [120]. As psychotic episodes are related with increased neuroinflammation and activated microglia [120, 121], it can be hypothesized that pro-inflammatory cytokines may be modulating BDNF mRNA expression via interaction of NF-κB or CREB transcription factors. It is known that schizophrenia patients have upregulated genes for inflammatory cytokines [122, 123], and downregulated Bdnf gene transcription [124, 125]. Neuroinflammation could be the key factor for the decrease of Bdnf gene expression in schizophrenic patients, as pro-inflammatory cytokines increase methylation of Bdnf gene, leading to a decrease of CREB binding to the specific Bdnf site.

Remarkably, drug treatment that is effective in controlling disease progression in schizophrenic patients can have diverse effects on peripheral BDNF protein levels [126–128]. In addition, some studies suggest that baseline BDNF levels in schizophrenia patients might reflect the susceptibility towards available drug therapies [129, 130]. Clearly, the interaction between neuroinflammation, BDNF levels, and treatment response should be better understood, as this could lead to identification of new targets for improved therapies.

## BDNF in Neurodegenerative Disorders

Despite research on neurodegenerative disorders has been increasing exponentially, there are still gaps in our knowledge on the etiology, onset and progression of most neurodegenerative diseases. Treatment is usually restricted to mitigation of the symptoms, rather than cure or delay of progression. Diagnosis of neurodegenerative diseases is usually based on subjective cognitive tests in combination with neuroimaging [131–133], but the current techniques are not able to successfully diagnose these diseases in their earlier stages, when the pathology is already present but does not cause symptoms yet. Attempts to discover new biomarkers for the diagnosis of early stages of the disease are ongoing [134–136]. Possibly, BDNF could qualify as such a biomarker.

In the following sections, we will discuss the role of BDNF in neurodegenerative disorders, focusing of Alzheimer’s disease, Parkinson’s disease, and epilepsy. We will also address the possible use of BDNF as a biomarker for diagnosis. In addition, we describe the role of neuroinflammation in the development of these diseases and explain how BDNF can help the brain to cope with inflammation.

### Alzheimer’s Disease

AD is characterized by a progressive loss of neurons in the brain, leading to impairment in memory and general cognition. Hallmarks of AD pathology are deposition of amyloid-β plaques in the extracellular matrix, formation of tau-phosphorylated neurofibrillary tangles within the cell and neuritic plaques. Tangles and plaques disrupt the signaling activity of neurons, eventually leading to neuronal apoptosis. As the disease progress, the axonal transport is constantly reduced, and the general function of neurons is impaired. These changes decrease BDNF axonal transport, resulting in reduced availability of BDNF within the synaptic cleft and consequently diminished signaling through TrkB receptors [137, 138]. BDNF mRNA and protein levels are also reduced in cognition-related structures such as hippocampus and frontal cortex, which corroborates BDNF depletion to be involved in the cognitive deficit leading to AD dementia [139]. BDNF levels are also reduced in plasma of patients with mild cognitive impairment (MCI) [140] and AD [141]. AD patients with higher serum concentrations of BDNF showed less cognitive decline after 1 year; this effect was more pronounced in the more severe stages of the disease [142, 143]. These studies suggest that BDNF might be a predictor for the rate of disease progression in AD.

Neuroinflammation is a key factor in the development and progression of AD [144, 145]. Amyloid-β deposits induce pro-inflammatory activation of microglia which might be an effort of microglia to mitigate the antigen-related damage [146]. Some studies have reported inflammatory challenges as an associated risk factor for the development of dementia-related symptoms, as they show increased pro-inflammatory cytokines levels [147, 148]. On the other hand, treatment with antiinflammatory medication tends to mitigate cognitive impairment in animal models of amyloid-β injection [149, 150]. Interestingly, PET imaging studies have shown that subjects with high amounts of amyloid-β, but no dementia, had decreased microglia activation [151–153], indicating a fundamental participation of microglia in the development and progression of AD.

There is no clear evidence on how effective BDNF can be in controlling neuroinflammation-dependent progression of the disease. Prakash shows that rats injected with Aβ in the hippocampus have a remarkable decrease in BDNF and increased TNF-α, IL-6, and caspase-3 protein levels 3 weeks after intracerebroventricular injection. These changes were associated with inability lack of memory retention in Morris Water Maze test [154]. Another study confirmed the increase in pro-inflammatory cytokines and reduced antiinflammatory cytokines and BDNF protein levels and gene expression after Aβ_1–42_ injection [155]. In humans, two studies have shown an increase in pro-inflammatory cytokines and a decrease in BDNF levels in the serum of early- and late-onset AD, although there was no correlation with pro-inflammatory and neurotrophin results in both studies [141, 156].

It is known, however, that pro-inflammatory cytokines—especially IL-1β—can cause downregulation of BDNF expression in cognition-related brain structures, such as the hippocampus [157, 158]. As these cytokines are upregulated in AD, a decrease of BDNF levels is expected to occur in such brain structures, leading to a decrease in survival signaling and, consequently, neuronal death. Moreover, increase in hyper-phosphorylated tau impairs anterograde transport of BDNF to the axon, further decreasing BDNF signaling in the synaptic cleft. As AD is a disease that inflicts several different physiological effects in both the internal and external milieus of the brain, efficient analysis of the role of BDNF in these processes is challenging and the overall effect of BDNF on brain function may be variable.

### Parkinson’s Disease

PD is characterized by movement impairment (bradykinesia, tremors, and rigidity), often combined with mild cognitive symptoms (decreased attention, executive function, memory) and mood disturbances (apathy, aggressiveness, anhedonia, depression) [159, 160]. The onset of PD is caused by the formation of aggregated α-synuclein plaques (i.e., Lewy bodies) in the substantia nigra pars compacta, leading to a progressive loss of dopaminergic neurons [161]. It is estimated that clinical symptoms start to appear when more than 50% of the neurons in the substantia nigra are already lost [162]. Movement symptoms are usually the first signs leading to the diagnosis of PD. As the disease progresses, more brain structures become affected by the neuronal loss and non-motor symptoms become evident [161, 163]. The severity of symptoms increases as the disease progresses and as a result, the patient loses gradually independence until patients become highly dependent on caregiver support in the late stages of the disease.

PD patients have lower concentrations of BDNF mRNA and protein in the substantia nigra pars compacta than healthy controls [164, 165]. Neurons with the lowest BDNF levels were suggested to be most prone to injury. Porritt and colleagues demonstrated that local inhibition of the production of BDNF with an antisense oligonucleotide leads to a significant loss of dopaminergic neurons in the substantia nigra pars compacta of rats, which suggests that BDNF has an important role in neuronal survival [166]. In contrast to the aforementioned studies, some reports describe that the BDNF levels in serum are increased in PD patients, especially in moderate to severe stages of the disease [167, 168]. This could mean that the CNS tries to cope with the loss of neurons by increasing BDNF production, resulting in enhanced serum levels of the protein. However, there is no direct evidence that supports this hypothesis.

The onset and progression of PD are also associated with neuroinflammation. Several studies in animal models of PD have reported increased microglial activation and pro-inflammatory cytokines [169, 170]. In humans, PD is associated with neuroinflammation in both postmortem [171] and in vivo analysis [172, 173]. Sawada and colleagues have found a remarkable increase of microglial cells in the hippocampus, amygdala, and entorhinal cortex of PD patients, which was associated with a decrease of BDNF mRNA expression and increased IL-6 in those regions [174]. Nagatsu has shown increased levels of IL-1β, IL-2, IL-6, and TNF-α in the striatum of PD patients, associated with a decreased BDNF protein levels in the same structure. Aggregated α-synuclein can induce an acute, local neuroinflammatory process in PD-associated brain structures, which suppresses BDNF expression and reduces BDNF protein levels. However, there is no evidence on how changes in BDNF levels in de brain affect the progression of PD and further analysis of the interaction between pro-inflammatory cytokines and BDNF is therefore necessary.

### Epilepsy

Epilepsy patients are affected by seemingly unprovoked seizures but usually also suffer from significant mood and cognitive changes [175]. The symptoms of epilepsy are induced by a disarray of excitatory neuronal connections, generating deregulated firing, or lack of inhibition of excitatory neurons. Although there are several treatment strategies to reduce seizures, 30% of the patients show little to no response to common antiepileptic drugs.

Seizures have been associated with an increased expression of several neurotrophic-related genes, including transcription factors [176], neuropeptides [177], and growth factors [178]. Two recent reports have shown that BDNF gene expression is increased in the hippocampus and temporal cortex of temporal lobe epilepsy patients [179, 180]. Seizures also increased hippocampal and cortical BDNF protein levels in animal model of epilepsy [181–183]. BDNF seems to be involved in epileptogenesis by regulating several signaling pathways within excitatory neurons, increasing Ca^2+^ signaling and glutamate expression [13, 184]. Overexpression of BDNF may also contribute to the epilepsy-induced cortical network deregulation by increasing even further the plasticity and dendritic branching signaling and causing an overexcitement state of glutamatergic neurons, thus reducing seizure threshold [185]. This creates a positive feedback loop: as upregulated glutamate neurotransmitter increases BDNF signaling and expression, it further increases glutamate signaling. Other studies have shown that transient inhibition of the BDNF receptor TrkB after seizure induction prevents the development of temporal lobe epilepsy [186, 187]. These findings indicate that BDNF is intimately related to the pathogenesis of epilepsy, and new therapeutic methods should take BDNF into consideration. However, it is worth noting that BDNF may also play a part in protecting neurons against harmful stimuli; therefore, treatment aiming to reduce BDNF levels as a whole should be carefully considered.

In epilepsy, chronic seizures lead to excitotoxicity and neuronal apoptosis with associated gliosis [188]. Studies in animal models have reported that seizures are associated with increased activation of microglia, especially of the M1 subtype [189], and an increased release of pro-inflammatory mediators [190, 191]. Although studies that link BDNF with neuroinflammation in epilepsy are lacking, a hypothesis for such a link can be formulated based on existing knowledge. In healthy conditions, BDNF signaling induces the activation of transcription factor NF-κB, which in turn induces de novo expression of the Bdnf gene [26, 192]. It is possible that overexpression of BDNF leads to an inflammatory response mediated by NF-κB, leading to astrocyte activation, increased production of cytokines and neurotoxic reactive oxygen species, and local recruitment of activated microglia [193]. Activated pro-inflammatory microglia are known to elicit apoptotic response after chronic stimulation, which causes neurotoxicity and further neuronal death. As BDNF is overexpressed at the onset of seizures [194], there is also an increase in BDNF-mediated glutamate signaling, contributing to the systemic neuronal imbalance. Although there are several players involved in the development of seizures, BDNF might be an important factor in modulating the disease. BDNF inducing, or being induced, by NF-κB also highlights the importance of neuroinflammation in modulating BDNF-dependent regulation. However, there is a need for further researches to better understand the role of BDNF in epilepsy, especially regarding its role in modulation of inflammatory processes.

## BDNF a Potential Therapy for Brain Pathologies

BDNF has been regarded as a possible biomarker for monitoring the onset, progression, and treatment of brain pathologies. However, recent studies suggest that BDNF may also be a potential target for new treatment strategies. Some promising results have been published from studies showing that therapeutic use of BDNF can, directly or indirectly, modulate changes within the brain [195]. An important finding is that peripheral BDNF is able to cross the blood-brain barrier [196], which is a prerequisite for BDNF-related therapy. However, the rate at which BDNF is taken up by the brain has not been quantified yet. Nonetheless, experimental therapy has been investigated in vivo and in vitro with promising results in animal models of AD [197], PD [198], and MDD [199] (a comprehensive review on BDNF drug delivery in brain pathologies and current stage of clinical trial can be found in [200]). For example, BDNF infusion into the hippocampus of adult rats was able to increase neurogenesis and regional neuronal activity [201]. Delivery of the BDNF mimetic 7-8-dihydroxiflavone is able to revert cognitive deficits in an AD animal model [202]. Also, gene transfection of BDNF into a 6-hydroxydopamine-induced unilateral lesion in the striatum was able to revert motor deficits in this animal model of PD [203].

The current need for improved treatments for brain disorders is pressing, and although large amounts of resources are spent on new therapies, few have been able to bring the desired results. The initial findings suggest that BDNF may be more than a biomarker for brain disorders; it may also become a possible target for the treatment of brain disorders. However, there is still a need for more information about the pharmacological features of BDNF-based substances in order to develop new treatments.

BDNF levels are activity-dependent, which means that the expression of BDNF changes under positive (e.g., physical activity, cognitive enhancement) and negative (e.g., obesity, sedentarism) behavioral and environmental stimuli. Several studies on both humans and animals show that physical activity, cognitive stimulation, and a balanced diet can stimulate BDNF expression [204–207]. Physical activity is the best studied positive factor for stimulation of BDNF expression. Thus, exercise can act as an inductor of neuronal plasticity, neurogenesis and neuronal survival [208, 209]. Several studies in both animals and humans have assessed the effects of physical activity on BDNF levels in psychiatric [210–213] and neurodegenerative diseases [214–217]. These studies indeed indicated that physical activity augments neuronal protection in brain disorders by stimulating BDNF expression. However, there are still some questions regarding the required time of the physical intervention, the optimal type of exercise (i.e., strength, endurance, aerobic exercises). Although less studied, diet can also provoke changes BDNF levels in physiological conditions. BDNF is known to affect the regulation of feeding and energy metabolism [218–221]. Decreased levels of BDNF were found in subjects consuming diets with high sugar and fat [222, 223]. On the other hand, dietary restriction (i.e., the maintenance of a balanced diet) or addition of supplementary substances (e.g., omega-3 fatty acids; resveratrol) can induce an increase in BDNF levels and reduce cognitive impairment in animal models [224–226]. It is not clear yet how large the effect of dietary management on BDNF protein levels in psychiatric or neurodegenerative disorders, but it is known that BDNF affects—and is affected by—dietary behavior. More research is needed in order to better understand the dietary influence on brain disorders.

Lifestyle changes that modulate BDNF levels in the brain may prove capable to control symptoms and delay progression in brain pathologies and thus might become cheap and easily accessible (adjuvant) treatment for these pathologies in the near future. However, a possible concern about such therapies is the compliance of the patients with the treatment. Most of behavioral therapies are based on long periods of treatment with a relatively slow improvement when compared with medications. This may reduce the motivation of patients to continue treatment, especially because most psychiatric and neurodegenerative disorders can be accompanied by mood symptoms (e.g., anhedonia, hopelessness, aggressiveness). Therefore, slow-acting lifestyle therapies could yield better results if they are combined with fast-acting pharmacological interventions.

## Concluding Remarks

BDNF is involved in several processes that are essential for the optimal functioning of the brain. Several studies report altered brain and plasma BDNF levels in patients with various brain pathologies. However, the pathophysiological mechanisms that underlie these changes are still not fully understood yet. There is also no conclusive evidence that can discriminate whether changes in BDNF levels are a causative or the consequence of the disease onset. It is challenging to prove a causative role of BDNF in psychiatric and neurodegenerative disorders. Some circumstantial evidence suggest such a causative role for BDNF. Human populations with a genetic variant that decreases the BDNF concentration appear to be more susceptible to psychiatric disorders. However, it should be noted that BDNF is highly affected by environmental changes, which in turn may affect genetic findings. In animals, injection of BDNF in the hippocampus was shown to cause a decrease of depressive-like behavior. However, it remains unclear how these findings translate to the clinical situation, as current disease models are not able to properly reproduce complex human disorders, because they usually only mimic part of the symptoms (i.e., low predictive validity). Direct translation of the animal findings to humans would imply an intervention by intrathecal BDNF injection in the brain or spinal cord of patients. However, such an intervention would be highly invasive and the success would depend on the ability of BDNF to migrate to the target region. Alternatively, interventions that aim to stimulate the production of BDNF could be prospectively investigated in patients with low BDNF levels, either due to a genetic predisposition or environmental factors. BDNF levels should be monitored repetitively to determine if changes in BDNF levels precede alleviation of symptoms or vice versa. In a population with a genetic predisposition for low BDNF levels, preventive intervention with a BDNF stimulating treatment could also be considered, but this would require a large study population and a long follow-up time.

Evidence from preclinical studies and clinical trials suggests that treatment strategies aiming to increase brain BDNF levels could have a beneficial effect on many brain disorders. In this respect, epilepsy is an exception as it is associated with increased levels of BDNF. At present, pharmacological intervention by administration of exogenous BDNF is still challenging, but lifestyle changes could provoke the desired effect. Environmental and physiological stimuli, such as physical activity, social interactions, and sensory and cognitive stimuli, are powerful modifiers of neurotrophin levels, including BDNF levels, and have been shown to alleviate symptoms of brain pathologies in both humans and animal models. Plasma BDNF can be reliably assayed, and samples are easily collected and therefore BDNF has been proposed as a potential peripheral biomarker for the assessment of the status of the brain and the efficacy of treatment. However, discrepancies between brain and plasma BDNF alterations have been observed and need to be further elucidated before plasma BDNF can qualify as a suitable biomarker.

Neuroinflammation is regulated by factors that are also involved in modulation of BDNF expression. Both neuroinflammation and altered BDNF expression are common phenomena in many brain disorders. Remarkably, there are only few studies that have investigated the link between BDNF and neuroinflammation. Better understanding of the interaction between BDNF and neuroinflammation could open new ways for therapy management and could facilitate the development of new therapeutic strategies for brain diseases.
